# Maternal sleep quality during pregnancy is associated with neonatal auditory ERPs

**DOI:** 10.1038/s41598-020-64160-8

**Published:** 2020-04-29

**Authors:** Maria Lavonius, Henry Railo, Linnea Karlsson, Valtteri Wikström, Jetro J. Tuulari, Noora M. Scheinin, E. Juulia Paavonen, Päivi Polo-Kantola, Hasse Karlsson, Minna Huotilainen

**Affiliations:** 1 FinnBrain Birth Cohort Study, Turku Brain and Mind Center, Institute of Clinical Medicine, University of Turku, Turku, Finland; 2 Department of Clinical Neurophysiology, University of Turku and Turku University Hospital, Turku, Finland; 3 Department of Psychology, University of Turku, Turku, Finland; 4 Department of Child Psychiatry, University of Turku and Turku University Hospital, Turku, Finland; 5 Cognitive Brain Research Unit, and CICERO Learning Network, Faculty of Educational Sciences, University of Helsinki, Helsinki, Finland; 6 Department of Psychiatry, University of Turku and Turku University Hospital, Turku, Finland; 7 National Institute for Health and Welfare, Helsinki, Finland; 8 Department of Obstetrics and Gynecology, University of Turku and Turku University Hospital, Turku, Finland; 9 Sleep Research Center, Department of Physiology, University of Turku, Turku, Finland

**Keywords:** Sleep, Attention, Sensory processing

## Abstract

Poor maternal sleep quality during pregnancy may act as a prenatal stress factor for the fetus and associate with neonate neurocognition, for example via fetal programming. The impacts of worsened maternal sleep on neonatal development and, more specifically on neonatal auditory brain responses, have not been studied. A total of 155 mother-neonate dyads drawn from the FinnBrain Birth Cohort Study participated in our study including maternal self-report questionnaires on sleep at gestational week 24 and an event-related potential (ERP) measurement among 1-2-day-old neonates. For sleep quality assessment, the Basic Nordic Sleep Questionnaire (BNSQ) was used and calculated scores for (1) insomnia, (2) subjective sleep loss and (3) sleepiness were formed and applied in the analyses. In the auditory ERP protocol, three emotionally uttered pseudo words (in *happy*, *angry* and *sad* valence) were presented among neutrally uttered pseudo words. To study the relations between prenatal maternal sleep quality and auditory emotion-related ERP responses, mixed-effects regression models were computed for early (100–200 ms) and late (300–500 ms) ERP response time-windows. All of the selected BNSQ scores were associated with neonatal ERP responses for *happy* and *angry* emotion stimuli (sleep loss and sleepiness in the early, and insomnia, sleep loss and sleepiness in the late time-window). For *sad* stimuli, only maternal sleep loss predicted the neonatal ERP response in the late time-window, likely because the overall ERP was weakest in the *sad* condition. We conclude that maternal sleep quality during pregnancy is associated with changes in neonatal auditory ERP responses.

## Introduction

Sufficient sleep is vital for health^1^. Poor maternal sleep quality during pregnancy may influence development and health of the neonate^2,3^. For instance, decreased sleep quality during pregnancy has been associated with higher levels of proinflammatory cytokines^4^ and increased probability of neonatal prematurity^2^, which may increase risk for lifelong neurocognitive difficulties. It has been reported that maternal sleep quality during the prenatal period – at least in the context of depression - is linked e.g. with the characteristics of the neonate’s observed sleep^5^. Thus, there is a need to investigate further the possible associations between the quality of maternal prenatal sleep and neonatal brain function.

### Prenatal factors and programming of fetal development

Psychosocial stress during pregnancy reportedly affects the programming of the fetus leading to altered predisposition to later health outcomes^6–8^. The *Developmental Origins of Health and Disease* (DOHaD) hypothesis, based on findings in a Dutch famine cohort study^9^, suggests that early-life (including prenatal) stress exposure shapes health outcomes and risk factors for chronic morbidity over the lifespan^8^. As reviewed by Abbott *et al*., various different factors can induce psychological distress, including negative life events and psychiatric symptoms^10^. Maternal psychological distress during pregnancy is reportedly associated with neurodevelopmental outcomes of the offspring, such as emotion and behavior regulation^11,12^. However, maternal sleep has been considerably less studied within the framework of prenatal stress exposures than, for example, maternal psychiatric symptoms.

Prenatal exposure to maternal stress may contribute to regulation problems of behavior and mental health of offspring via prenatal programming of the hypothalamus-pituitary-adrenal (HPA) axis^13,14^. However, while maternal glucocorticoid levels are linked with fetal growth, evidence for maternal emotional well-being associating with HPA axis activity is scarce^8^. Therefore, other mechanisms should be considered especially as psychosocial distress is broadly and complexly related to changes in e.g. glucocorticoid levels, immune system, sympathetic nervous system, and gut microbiota^15^. Finally, it should be noted that the neonatal neurodevelopment consequences followed by maternal distress are modulated by the genotype of the offspring^8^.

### Sleep during pregnancy

Although decreased maternal sleep quality is a common characteristic during pregnancy^16^ and a potential prenatal stress factor, the relations between maternal sleep characteristics during pregnancy and fetal or neonatal outcomes are still inadequately understood^17^. Sleep loss during pregnancy, especially when of chronic nature, may be comprehended as a physiological stress factor *per se* and is also often related to experienced stress^16^. It has been suggested that pathophysiological mechanisms possibly linking poor maternal sleep quality to later offspring health might affiliate especially with the HPA axis and an increased inflammatory reaction^16^. Sleep deficiency can be caused by shortened sleep duration, insufficient sleep or insomnia. Even in early pregnancy, 28-38% of women may suffer from inadequate sleep^18^, and subjectively reported sleep quality decline may manifest during any trimester^19^. Advancing from the second to the final trimester, sleep quality tends to decrease further^20^, with incremental nocturnal awakenings, difficulties in falling asleep and too-early awakenings^21^. In a Finnish population, approximately 12% of women reported either shortened sleep duration or significant insomnia at 32 weeks of gestation^22^.

### Neonatal ERPs

Auditory event-related potentials (ERPs) extracted from the electroencephalogram (EEG) have long been utilized to investigate change-detection processing and auditory discrimination in neonates [e.g.^23^]. Already in neonates, the generators of ERP responses related to change-detection are functional^24^ and ERPs can be elicited without recording paralleling behavioral responses as opposed to older children. Kushnerenko *et al*. estimated that neonates seem to be able to detect a novelty in acoustic stimuli but with a longer latency compared to adults^25^. Mismatch negativity (MMN) response reflects an auditory change-detection process and can be elicited in adults by unattended auditory stimuli^26^ in an oddball paradigm featuring frequent standard and occasional deviant stimuli. Previously, a multi-feature MMN paradigm including three rarely occurring emotionally valenced speech stimuli was developed for analyzing processing of emotional speech content and attention allocation^27^. This paradigm was developed to aim at potential applications in studying pathologies, such as alexithymia, autism spectrum and attention disorders, in children^27^. In a study investigating simple pitch change-detection in an oddball paradigm, the MMN response was identified in 75% of neonates, and the amplitude and latency were shown to be relatively stable at group level albeit responses produced considerable inter-subject variability^28^. In addition to the MMN response, a later-latency response often termed P3-type response has been observed in the oddball paradigm for large acoustic changes in neonates^28^, suggested to be a precursor of future processes of involuntary attention. These two responses in infancy have been linked to later cognitive development, like language and executive skills^29,30^. We wanted to record brain responses reflecting perceptive, attentional and memory-related functions to emotional sounds in infants in order to map the cognitive processes related to the development of these relevant skills during the fetal period.

Based on previous studies on adult samples, fetal stress exposure is related to later cognitive performance i.e. lower intellectual ability^31^. While ERPs have their advantages in assessing change-detection, attention allocation and other temporally measured cognitive processes in neonates, few studies have investigated neonatal ERP responses in the context of prenatal maternal stress exposure. Consequences of maternal anxiety during the perinatal^32^ and prenatal^33,34^ periods on neonatal cognition have been studied previously with neuropsychological measures. The existing data suggest that fetal exposure to maternal prenatal stress, in the form of maternal anxiety, is related to less prominent frontal negative slow wave amplitudes in response to mother’s voice^32^ and increased offspring’s neurocognitive responses in an oddball paradigm^34^, prompting a theory for anxiety-driven neurophysiological differences in attention allocation in stress-exposed neonates. In addition to maternal anxiety, sleep adversities may influence fetal programming, but the independent relations between maternal sleep and neurodevelopmental measures in neonates have remained undiscovered.

## The present study and the hypothesis

In this study, we investigated whether prenatal maternal sleep quality, as a marker of prenatal environmental exposure, is related to neonatal auditory event-related potentials (ERPs) in the context of emotional auditory stimuli. In this protocol, three emotionally uttered pseudo words (in *happy*, *angry* and *sad* valence) were presented to neonates among neutrally uttered pseudo words during an EEG recording. We anticipated that neonates exhibit ERP responses of different magnitudes in regard to emotional valence of the auditory stimuli and that maternal sleep characteristics are associated with the neonatal ERPs to the emotional sounds.

## Materials and methods

### Participants

This study sample was drawn from the FinnBrain Birth Cohort Study (N = 3808 families) from Turku region in Southwest Finland and the Åland Islands (www.finnbrain.fi^35^). Families had originally been recruited to the FinnBrain Study at the first trimester ultrasound visit [around gestational week (gwk) 12] if they met the inclusion criteria of having sufficient knowledge of Finnish or Swedish languages and a verified ongoing pregnancy. The Ethics Committee of the Hospital District of Southwest Finland had approved the study (ETMK 57/180/2011) and study methods were carried out in accordance with their guidelines.

The current study sample consisted of 155 mother-neonate dyads recruited by a researcher ( ML) for an EEG recording at the maternity wards of Turku University Hospital. This sample comprises of Birth Cohort participants who gave birth between 2013 and 2015. Recruitment took place on average 1-2 days after birth, the youngest neonate being 7 hours and the oldest 5 days of age during the EEG recording. Parents received information on the study procedure prior to data collection and a written consent was collected. Exclusion criteria for the EEG recording included birth at <gwk 36 + 0, neonate birth weight <1800 g, any severe deformity or developmental disorder, known hearing defect (routinely screened for all infants) or neonate’s medical follow-up based on mother’s severe illness.

**Figure 1:**
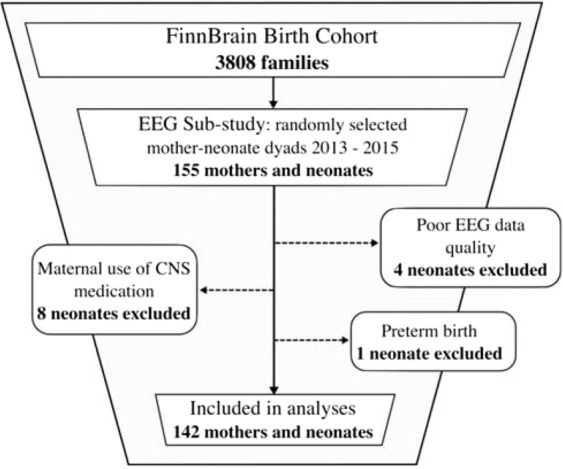
Inclusion and exclusion of mother-neonate dyads.

Data of four neonates were omitted from the EEG analyses for excess artefact and poor data quality. Data of further eight neonates were excluded based on maternal use of central nervous system (CNS) affecting medication during pregnancy and of one neonate for preterm birth (born at 34.4 gwk). Therefore, data of 142 mother-neonate dyads remained for the statistical analyses (Fig. 1). In this final study sample five families were Swedish-speaking. The characteristic variables for mother-neonate dyads are presented in Table 1.

**Table 1:** Demographics of mothers and neonates included in the analyses (n = 142).

	Mean ± SD or % (n)	Range
**Mother**		
Age (years)^a^	30.8 ± 4.7	18.9–42.2
BMI (kg/m^2^)^b^	24.4 ± 4.6	17.1–45.6
Nulliparous^c^	58.3% (74)	
Multiparous^c^	41.7% (53)	
BNSQ insomnia^d^ at gwk 24	7.7 ± 2.1	4–12
BNSQ sleep loss^e^ at gwk 24	1.0 ± 1.0	−1.5–4.0
BNSQ sleepiness^d^ at gwk 24	5.5 ± 2.2	2–10
EPDS^d^ at gwk 24	5.1 ± 4.9	0–25
**Neonate**		
Sex (girls/boys)	47.9% (68)/52.1% (74)	
Gestational weeks at birth	40.0 ± 1.2	36.1–42.3
Birth weight (g)	3579 ± 443	2635–5470
Age at EEG recording (days)	1.4 ± 1.0	0–5

Note: BNSQ = Basic Nordic Sleep Questionnaire; EPDS = Edinburgh Postnatal Depression Scale; gwk = gestational week.

Some data were missing due to attrition in questionnaire and register data acquisition.

Data available for ^a^n = 108, ^b^n = 140, ^c^n = 127, ^d^n = 121, ^e^n = 119.

### Procedures

This study comprised of questionnaire data collected during pregnancy and ERP recordings on neonates. As administered by the FinnBrain Birth Cohort Study, prenatal questionnaires were collected at three time-points: 14, 24 and 34 gwk, of which data of gwk 24 was used in this study. The questionnaires could be filled online or sent via mail. The Finnish Medical Birth Register kept by the Finnish National Institute for Health and Welfare (www.thl.fi) was used to provide information of maternal age (years), parity (number, analyzed as nullipara/multipara) and body mass index (BMI; kg/m^2^) before pregnancy, obstetrical and pediatric diagnoses and medication usage during pregnancy, as well as gestation length (days), neonate birth weight (grams) and child sex. Regarding prescribed medications, antidepressants, anticonvulsants, benzodiazepines and benzodiazepine-like sleep medication were extracted and used as exclusion of the participants from further analyses. After delivery, an EEG recording session was scheduled at the maternity wards. The methods used in this study did not expose the neonates to any medical risks.

## Measures

### Maternal sleep quality during pregnancy

The self-report Basic Nordic Sleep Questionnaire (BNSQ) was used to assess maternal sleep during pregnancy at 24 gwk. This questionnaire is reportedly a valid tool used widely especially in Nordic populations^36^. The BNSQ consists of 27 items in 21 different questions of which 7 questions were adopted in this current study: difficulty falling asleep, nocturnal awakenings, too-early morning awakenings, sleep duration (estimated in minutes), preferred sleep duration (minutes), morning sleepiness and daytime sleepiness. The evaluation of sleep quality was assessed over the previous three months^21^. Of these questions, three scores were formed: (1) insomnia score (replies in questions of difficulty falling asleep, nocturnal awakenings and too-early morning awakenings combined; Cronbach’s Alpha = 0.522), (2) sleep loss score (actual sleep duration subtracted from preferred sleep duration) and (3) sleepiness score (morning and daytime sleepiness combined; Cronbach’s Alpha = 0.843).

### Maternal depressive symptoms during pregnancy

To control for the potential influence of maternal depressive symptoms, the Edinburgh Postantal Depression Scale (EPDS) was used. EPDS is among the most frequently used self-report scales to screen postpartum depression^37,38^, also valid for screening depressive symptoms during pregnancy^39^. It consists of 10 questions (rated between 0 and 3 on a 4-point Likert scale) providing a total score range between 0 and 30. In this general population-based sample, a continuous total EPDS sum score was used.

### Neonatal ERP recording and data analysis

In order to achieve a calm sleep phase and to minimize restlessness during sleep, the EEG measurement was conducted within two hours after feeding of the neonate. Of neonates, 24% were tested during the forenoon and the rest during the afternoon. A parent was present during data collection for 16 neonates (11%). Should the neonate be awake after putting on an EEG-headpiece (ActiCap electrode cap by EASYCAP, Germany) and applying electrode gel (Signa Gel, Parker Laboratories, Inc. USA), he/she was assisted to sleep by giving a pacifier (used for 37% of neonates during recording) or mild glucose solution if needed. The neonates were sleeping on the left side, the right ear facing upwards. EEG was recorded with 16 channels (International 10–20 system) using BrainVision Quickamp amplifier (BrainProducts, Germany). The reference and ground electrodes were located on the forehead. Sampling frequency was 500 Hz. Alertness of the neonate was evaluated visually by a researcher. An auditory sound stimulus including three emotionally valenced pseudo words was presented twice from a loudspeaker situated approximately 1 meter from the neonate and at a standard volume of 60 dB. Any prolonged crying ended the recording at any moment. Of all neonates, 17 (12%) were fully but calmly awake and 4 (3%) partly awake during recording. The measurement session duration was approximately 45 minutes.

We used emotionally uttered pseudo words of the multi-feature paradigm presented in full detail by Kostilainen *et al*.^40^. The ERP responses were recorded with a stimulation paradigm including a standard bi-syllabic /ta-ta/ pseudo word stimulus (336 ms total duration, 46% probability, 400 trials in total recording), four types of linguistically relevant deviant pseudo words (changes in vowel duration, vowel identity, intensity and frequency), and three emotionally uttered /ta-ta/ pseudo words (*happy*, *angry*, *sad*: total durations 388 ms, 337 ms and 436 ms respectively, 3% probability and 24 trials in total recording for each emotion) that occurred infrequently among the stimulus streams. The stimuli were presented with an onset-to-onset time of 650 ms regardless of the duration of each stimulus.

**Figure 2:**
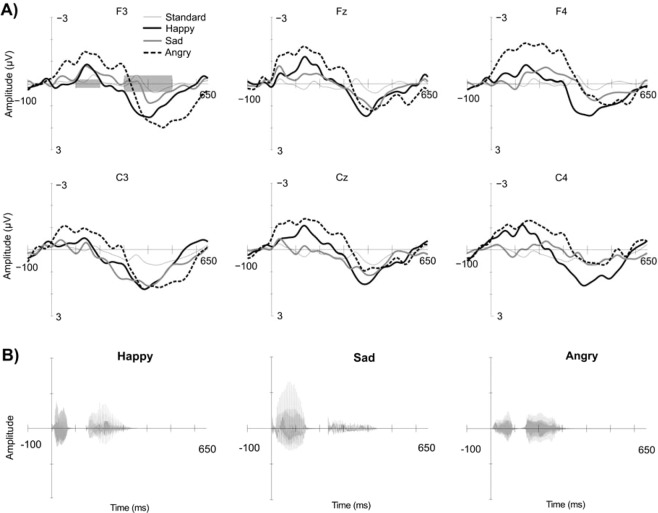
ERP waveforms. (**A**) The mean ERP amplitudes of the whole group of neonates for the three emotional stimuli and standard stimulus separately for six electrode locations. The two grey highlights at the timeline in upper left corner represent the two time-windows at 100-200 ms and 300-500 ms that were used in the analysis. (**B**) The sound waves of the emotional speech stimuli at the timeline.

EEG data was analyzed using Matlab R2014a, EEGlab v12.0.2.5b and in-house tool CBRUplugin v1.34b. Filtering was done applying a 1 Hz high pass filter and a 20 Hz low pass filter. 36-point low pass filter was performed with a transition band width of 3 Hz and 750-point high pass filter with a transition band width of 0.2 Hz. The EEG data was re-referenced using Oz electrode location. Electrodes F3, Fz, F4, C3, Cz and C4 were analyzed separately. An epoch length of −100 ms – 650 ms with a −100 ms baseline correction was used. Epochs yielding events larger than ± 150 μV were rejected. Eventually, data of four neonates with <50% of total epoch count remaining after rejection was excluded from further analyses. After rejection, the average number of trials remaining for each event were as follows: for happy 21.87 trials (3.05 SD), for sad 21.50 trials (3.40 SD) and for angry 21.92 trials (3.03 SD). Epoch for each trigger and neonate were averaged separately and mean EEG amplitudes (for electrode locations F3, Fz, F4 & C3, Cz, C4) were calculated for early (100-200 ms) and late (300-500 ms) time-windows (Fig. 2). Previous studies in this age group and using the exactly same stimuli show that evoked activity is present in these time-windows^40^. Finally, for statistical analyses, the three frontal and three central electrodes were averaged as one mean amplitude per neonate for each of the two time-windows.

### Statistical analyses

Statistical analyses were performed in using SPSS 24.0 (IBM Corp., Armonk, NY, USA) and R software. First, a descriptive analysis was computed with maternal age (years), BMI (kg/m^2^), sleep traits (BNSQ scales insomnia, sleep loss and sleepiness at gwk 24) and depressive symptoms (EPDS total score at gwk 24) as continuous variables and parity as a categorical variable (nulliparous or multiparous). Furthermore, neonate variables included in the descriptive analysis were gender as nominal, and birth weight (g), gestational age at birth (days) and age at EEG recording (days) as continuous variables. These variates are presented as means and standard deviations or percentages. Then, two-tailed one-sample t-tests were computed to inspect which mean EEG amplitudes differed from 0 μV in averaged electrode locations (F3, Fz, F4, C3, Cz and C4). Here our aim was to characterize how strong ERP deflections are produced. Next, correlation coefficients were calculated between demographic mother-neonate data, magnitudes of ERP amplitudes (for three emotional sounds in two sequential time-windows), three BNSQ scores and EPDS were computed in order to check for multicollinearity. Only up to moderate correlations were observed between the independent variables (r ranging from 0.237 to 0.447). Finally, to examine if maternal sleep quality predicts ERP amplitudes, linear mixed-effects regression models were fitted in R using the lme4 package. All the models had the following structure for fixed effects

$$\begin{array}{c}Sleep+Emotion+Window+Location[+Covariates]+Sleep\times Emotion+Sleep\times Window\\ +\,Emotion\times Window+Sleep\times Emotion\times Window\end{array}$$

and the following random effects

$$(Emotion+Window+Emotion\times Window)/Neonate$$

*Sleep* means one of the variables (sleep loss, sleepiness or insomnia). *Emotion* (happy, angry, sad), *Window* (happy, angry, sad), (indicating early or late time-window (early vs. late) and *Location* (indicating electrode location: F3, Fz, F4, C3, Cz or C4) are categorical variables. *Location* was included in the model as a predictor to explain variation across electrodes. *Covariates* includes the variables: neonates gestational age (days) and sex (boy, girl) and maternal BMI (kg/m^2^), EPDS, parity (nulliparous, parous) and age (years). All the models were fitted both with and without the *Covariates*.

The random-effect structure was chosen by trying the different combinations of Emotion, Window and Location and then selecting the one that gave the smallest AIC and BIC values. The regression coefficients corresponding to the associations between each sleep quality variable and ERP amplitudes for each emotion in each time window were extracted from the models by changing the reference levels of Emotion and Window appropriately.

## Results

The demographic characteristics of the study population are described in Table 1. ERP amplitudes were statistically significantly different from zero for the following emotional variants: early time-window *happy* and *angry* and late time-window *happy* (Table 2). The early time-window (at 100–200 ms) produced mean amplitudes of negative polarity and conversely, the late time-window (at 300–500 ms) of positive polarity.

**Table 2:** Mean ERP amplitudes and difference from 0 μV by two-tailed one sample T-tests (n = 142).

	mean (*μ*V)	SD	95% CI	t-value	P-value
Amplitude					
**Early time-window**					
happy	−0.79	4.32	−1.50–−0.07	−2.17	0.032
sad	−0.44	4.90	−1.25–0.37	−1.07	0.287
angry	−1.13	4.35	−1.86–−0.41	−3.10	0.002
**Late time-window**					
happy	0.94	4.42	0.20–1.67	2.53	0.013
sad	0.69	4.91	−0.13–1.50	1.67	0.098
angry	0.77	5.26	−0.10–1.64	1.74	0.083

### Mixed-effects regression models

**Figure 3:**
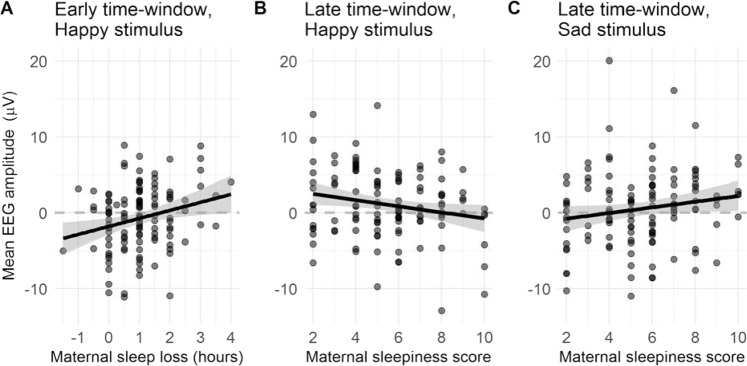
Results. Correlations between (**A**) maternal sleep loss (early time-window, happy stimulus), (**B**) maternal sleepiness (late time-window, happy stimulus), and (**C**) maternal sleepiness (late time-window, sad stimulus) and neonate average ERP amplitudes (averaged across all electrodes). The lines are simple linear fits across the data points, not the results of the mixed-effects models.

The results of the mixed-effects regression models showed that in the early time-window maternal sleep loss positively correlated with neonatal ERP responses to happy stimuli (ß = 1.06, 95% CI [0.29, 1.84], p = 0.008). That is, one hour increase in sleep loss was associated with 1.06 μV increase in the ERP amplitude in the early time-window to a happy stimulus. Note that because amplitudes are, on average negative in the early time-window, the positive correlation means that the ERP decreased as sleep loss increase. In the late time-window, maternal sleepiness correlated negatively with ERP amplitudes in response to happy (ß = −0.41 [−0.77, −0.05], p = 0.025) and positively to sad (ß = 0.37 [−0.03, 0.78], p = 0.068) stimuli (interaction between these: ß = 0.78 [0.25, 1.31], p = 0.004). Because mean amplitudes were positive in the late time-window, a negative correlation indicates that amplitude decreases. These associations are presented in Fig. 3.

To control for the possibility that the observed associations were confounded by differences in other factors, we ran further models that included maternal depressive symptoms, age, parity and BMI and neonate’s sex and gestational age. Two of the three correlations reported above remained statistically significant even after controlling for the variation in these confounding factors. In the early time-window sleep loss was associated with ERP amplitude to happy stimuli (ß = 1.01, 95% CI [0.22, 1.80], p = 0.012). In the late time-window, maternal sleepiness correlated negatively with ERP amplitudes in response to happy (ß = −0.50 [−0.87, −0.13], p = 0.0076). (The correlation between sleepiness and sad stimuli was ß = 0.28 [−0.17, 0.68], p = 0.17 when covariates were included in the model).

## Discussion

According to our hypothesis, fetal cognitive development and well-being, here reflected by neonatal ERP responses to emotional sounds, would associate with fetal stress factors, here reflected by maternal sleep quality. We anticipated that neonates exhibit ERP responses of different magnitudes depending on the exposure of distress as reflected by maternal sleep characteristics during pregnancy. Our results support the hypothesis that maternal sleep patterns predict variance in neonatal auditory ERP responses: maternal sleep loss and sleepiness were associated with neonatal ERP responses to *happy* and *sad* emotion stimuli. Specifically, maternal sleep loss and sleepiness were observed to reduce the amplitude of the neonates’ ERP response to a happy stimulus. In contrast, maternal sleepiness enhanced the infant’s response to sad stimuli. These results are important, since decrease in maternal sleep quality is common during pregnancy^16^ and very little is known on its relationship with offspring brain development. Because our results are correlations, one cannot draw conclusions about the causal relationship between the factors based on our study.

While it is possible that maternal sleep problems causally lead the infant to process emotional stimuli differently, it is also possible that the observed correlation reflects e.g. shared genetic factors. Early life stress exposure, e.g. in the form of maternal anxiety, has been linked to detectable developmental brain alterations in offspring, potentially via fetal programming [e.g.^44–47^]. Prenatal maternal sleep characteristics and disorders may also act as a physiological and psychosocial stress factors *per se*^16^ and potentially mediate adverse neonatal neurocognitive outcomes, although research has been scarce from this framework, so far. The combination of potential mechanisms yielding and mediating prenatal fetal programming is still inadequately understood. A plausible mechanism relates to maternal psychosocial stress and cortisol levels with alterations in maternal immune functioning^15,48^. Additionally, genetic traits, both independently and in interaction with environmental factors, may propose another mechanism mediating fetal programming. Regarding inherited sleep traits, parents and their children may share genetic variants that predispose to poor sleep [for a review, see^49^]. It can also be speculated that neonatal auditory brain responses, as markers of fetal neurocognitive functioning, might share similar origins in brain regulatory functions as maternal sleep characteristics, e.g. via shared genetic background.

Albeit a topic of great interest and with possible implications for neonatal health, we are not aware of any previous studies approaching the same study question in a similar population. Earlier studies have investigated the relations of prenatal maternal anxiety exposure and neonatal ERP responses produced by the voice stimuli of neonate’s mother and a strange woman^32^, and by a paradigm including repetitious standard, white noise and novel environmental-like sounds as deviant events^34^. These studies yielded associations between anxiety exposure and altered neonatal neurocognitive functioning. However, these previous studies approached the exposure of early life stress in the form of maternal anxiety instead of sleep quality. Here, we chose to measure prenatal maternal sleep quality as chronic sleep loss has been shown to link to reactions of stress-related HPA axis and immune system alterations [for a review see 16] and maternal insomnia is further associated with adverse fetal outcomes^2^.

The significance of neonatal brain responses to emotional speech content from the perspective of brain developmental measures are still poorly understood. In the paradigm used in our study, emotional stimuli have been shown to elicit more prominent ERP responses compared to standard and deviant stimuli^40^. Also, as previously reported^24^, the ERPs revealed relatively large interindividual variation. When interpreting the results, it should be noted that the number of stimuli per neonate was limited and the neonates presented with differential alertness levels during EEG recording. Regardless these reservations, it is noteworthy, that maternal sleep loss and sleepiness yielded statistically significant changes in neonatal ERP responses in our study, also after the inclusion of several important covariates (maternal depressive symptoms, age, parity and BMI and neonate’s sex and gestational age).

In our paradigm, we hypothesized that the earlier time-window produced a novelty-MMN type response and the late time-window a novelty-P3 type response. Here, the emotional stimulus of *sad* valence was observed to produce the smallest ERP response peaks among the three emotion variants and maternal sleep was related to opposite changes on ERP responses for *sad* stimulus compared to responses for *happy* stimuli. Consistent with previous findings and likely due to different acoustical features among the three emotional variants, we observed the strongest (negative) mean amplitude in the early time-window for the *angry* variant. The emotional stimulus *angry* includes stronger natural changes in acoustical timbre, stress and intensity at the first syllable compared to the *happy* and especially to the *sad* emotional stimuli, hence prompting the largest amplitudes at the early 100-200 ms time window in a young infant population of 202 infants^40^. In general, we wish to note that based on the present approach it is impossible to disentangle how emotional valence specifically contributes to ERP amplitudes. Our crucial claim is just that the stimuli differed with respect to emotional valence, although they obviously also differed with respect to acoustic features. Here, the strongest (positive) mean ERP amplitude peak was observed in the late time-window for the *happy* variant. Inhibitory brain circuit function and neurotransmitter balance may provide mechanisms that further explain the findings as to why our neonate sample produced statistically significant ERP responses to emotionally valenced pseudo word stimuli only for *happy* and *angry* in the early time-window and only for *happy* in the late time-window^41–43^. In our paradigm, neonates were presented with repetitive neutral pseudo words and, should maternal stress in form of poor sleep quality have interfered with the neonatal inhibition function, rare emotionally valenced deviant stimuli might have produced weaker ERP responses.

The second trimester sleep questionnaire (at gwk 24) was chosen for our analyses based on physiological grounds. As stated, sleep towards the end of pregnancy is easily affected by prenatal physiological symptoms and discomfort, and a large percentage of mothers experience disrupted sleep of some degree^21^. Thus, we anticipated that application of the mid-pregnancy data in mixed-effects regression analysis might allow a better variance of diverse sleep quality in mothers. Including the sleep quality measurement at gwk 14 could have lead for less variance, as the occurrence of sleep disturbances are still minor^50^. Furthermore, it is possible that sleep disturbances during the first phase of pregnancy may be of different origin and even resolve as pregnancy proceeds, thus not probably inducing long term stress. In addition, using the gwk 24 sleep data for our analyses, we considered that the time until the delivery, approximately 16 weeks, was long enough to induce long lasting stress to affect neonatal ERP responses, whereas if using the data from gwk 34, the variance of sleep disturbances between women would have probably been too small.

Depressive symptoms were included in our mixed-effects regression modelling explaining ERP responses, yet sleep scores remained as significant predictors over the EPDS score. As an adjusting variable in our mixed-effects regression models, neonate sex did not explain the variance in ERP responses. Despite this finding, we suggest including potential sex differences in any future investigation of neonatal processing of auditory emotional stimuli. Moreover, the follow-up of neonates will be an important next step in order to investigate the possible relevance of this EPR variation during early neurodevelopment.

### Strengths and limitations

We studied a sizable mother-neonate sample, and data of only four neonates out of 155 had to be excluded due to poor quality. Moreover, the mean age of the studied neonates was only 1.4 days, which uniquely emphasizes the effect of prenatal factors and minimizes the impact of varying postnatal environment. The recruitment of the FinnBrain Birth Cohort mothers to the ERP study was random at the maternity wards and e.g. psychiatric morbidity was not enriched among the mothers^35^. We used selected questions from BNSQ and formed insomnia and sleepiness scores instead of using entire questionnaire with separate items. This was feasible, as according to the validation study of the questionnaire, no strict rules for using the questionnaire are given. Furthermore, by using scores for insomnia and sleepiness symptoms instead of separate items of insomnia and sleepiness symptoms, we achieved more strength for the sleep variables and thus probably also found women with accumulated sleep disturbances.

Despite these strengths, our investigation had some limitations. First, BNSQ, despite being a validated tool^36^, is a self-report questionnaire and no objective sleep measures were used to collect data on maternal sleep quality during pregnancy. Second, due to the general population-based design and normative samples, we were unable to include any sleep disturbances, such as restless legs or breathing-related problems, to our analyses. Third, our study sample of mothers was relatively healthy at the time of their pregnancy^35^, and sleep quality in conjunction with e.g. psychiatric disorders should be addressed in future studies. Maternal use of psychotropic medication was an exclusion criterion which precludes further extrapolation of our findings to a population with major psychiatric disorders or pregnancy complications. Fourth, regarding the neonatal ERP measurement, the alertness level was not included in the analysis, and differences in alertness remains a potential confound. Previous research is somewhat inconsistent on whether the alertness level should be considered in neonatal ERP recordings^51–54^. In addition to alertness level, the role of neonatal brain maturation and the gestational age should be taken into consideration in auditory brain potential studies in neonates^55^. Here, we included neonates between gwks 36 and 42, a range that may predicate on varying maturational levels and polarity of neonatal ERP responses. Fifth, neonates who used a pacifier during ERP recording were not excluded from the analysis, which may have produced glossokinetic artifact. A high-pass EEG filter was introduced in data analysis to remove these artifacts. Also, what should be noted according to Kostilainen *et al*. is the potential challenge of the applied multi-feature paradigm, especially in a neonatal population, as the amount of presented auditory deviant stimuli is rather large and the presentation rate is quite fast^40^.

## Conclusion

Based on this novel study, we can conclude that maternal sleep characteristics are associated with neonatal auditory ERP responses. Interestingly, the direction of the association varied between the different sleep characteristics and emotional valence of the stimuli. This study adds to the growing body of evidence linking maternal well-being during pregnancy and neonatal neurocognitive performance. However, further investigation on these relations is called for. Especially the significance of the different neonatal ERP responses for later development remains to be evaluated by follow-up assessments.

## Data Availability

Data is available on request. The requests should be directed to the Scientific Board of the FinnBrain Birth Cohort Study (PI prof Hasse Karlsson, hasse.karlsson@utu.fi; co-PI Adj Prof Linnea Karlsson, linnea.karlsson@utu.fi). Any agreements of material transfer are made between the University of Turku (study site) and the possible recipient following a positive decision from the Cohort Scientific Board. Currently, the Finnish federal legislation/Personal Data Act on personal data protection prohibits data sharing into an open repository. According to the Finnish legislation, the study subjects should have provided a written informed consent on sharing the data for this specific purpose and for this specific repository. Unfortunately, at this stage of our cohort study, it is not possible to obtain new, complimentary consents from each subject. Thus, access to data is subject to scientific collaboration or other agreement between the Scientific Board of the Cohort and the University of Turku (study site).

## References

[1] Gilmour H (2013). Longitudinal trajectories of sleep duration in the general population. Health Rep..

[2] Felder JN, Baer RJ, Rand L, Jelliffe-Pawlowski LL, Prather AA (2017). Sleep Disorder Diagnosis During Pregnancy and Risk of Preterm Birth. Obstet Gynecol..

[3] Nodine PM, Matthews EE (2013). Common sleep disorders: Management strategies and pregnancy outcomes. J Midwifery Womens Health.

[4] Chang JJ, Pien GW, Duntley SP, Macones GA (2010). Sleep deprivation during pregnancy and maternal and fetal outcomes: is there a relationship?. Sleep Med Rev.

[5] Field T (2017). Sleep disturbances in depressed pregnant women and their newborns. Infant Behav Dev.

[6] Huizink AC, Robles de Medina PG, Mulder EJH, Visser GHA, Buitelaar JK (2003). Stress during pregnancy is associated with developmental outcome in infancy. J Child Psychol Psychiatry.

[7] Mulder EJH (2002). Prenatal maternal stress: effects on pregnancy and the (unborn) child. Early Hum Dev.

[8] O’Donnell KJ, Meaney MJ (2017). Fetal Origins of Mental Health: The Developmental Origins of Health and Disease Hypothesis. Am J Psychiatry.

[9] Susser E, Hoek HW, Brown A (1998). Neurodevelopmental disorders after prenatal famine: The story of the Dutch Famine Study. Am J Epidemiol.

[10] Abbott PW, Gumusoglu SB, Bittle J, Beversdorf DQ, Stevens HE (2018). Prenatal stress and genetic risk: How prenatal stress interacts with genetics to alter risk for psychiatric illness. Psychoneuroendocrinology..

[11] Glover V (2014). Maternal depression, anxiety and stress during pregnancy and child outcome; what needs to be done. Best Pract Res Clin Obstet Gynaecol.

[12] O’Donnell KJ, Glover V, Barker ED, O’Connor TG (2014). The persisting effect of maternal mood in pregnancy on childhood psychopathology. Dev Psychopathol..

[13] Van den Bergh BRH, Mulder EJH, Mennes M, Glover V (2005). Antenatal maternal anxiety and stress and the neurobehavioural development of the fetus and child: links and possible mechanisms. A review. Neurosci Biobehav Rev.

[14] Van den Bergh B. R. H. *et al*. Prenatal developmental origins of behavior and mental health: The influence of maternal stress in pregnancy. *Neurosci Biobehav Rev*, 10.1016/j.neubiorev.2017.07.003 (2017). 10.1016/j.neubiorev.2017.07.003 28757456

[15] Glover V (2015). Prenatal Stress and Its Effects on the Fetus and the Child: Possible Underlying Biological Mechanisms. Advances in neurobiology.

[16] Palagini L (2014). Chronic sleep loss during pregnancy as a determinant of stress: impact on pregnancy outcome. Sleep Med..

[17] Oyiengo D, Louis M, Hott B, Bourjeily G (2014). Sleep disorders in pregnancy. Clin Chest Med.

[18] Okun ML (2013). Prevalence of Sleep Deficiency in Early Gestation and its Associations with Stress and Depressive Symptoms. J Womens Health.

[19] Neau JP, Texier B, Ingrand P (2009). Sleep and vigilance disorders in pregnancy. Eur Neurol..

[20] Hayase M, Shimada M, Seki H (2014). Sleep quality and stress in women with pregnancy-induced hypertension and gestational diabetes mellitus. Women Birth.

[21] Polo-Kantola P, Aukia L, Karlsson H, Karlsson L, Paavonen EJ (2017). Sleep quality during pregnancy: associations with depressive and anxiety symptoms. Acta Obstet Gynecol Scand.

[22] Paavonen EJ (2017). Maternal and paternal sleep during pregnancy in the Child-sleep birth cohort. Sleep Med..

[23] Alho K, Sainio K, Sajaniemi N, Reinikainen K, Näätänen R (1990). Event-related brain potential of human newborns to pitch change of an acoustic stimulus. Electroencephalogr Clin Neurophysiol.

[24] Kushnerenko E (2002). Maturation of the auditory event-related potentials during the first year of life. Neuroreport..

[25] Kushnerenko E (2007). Processing acoustic change and novelty in newborn infants. Eur J Neurosci. [Internet].

[26] Alho K, Woods DL, Algazi A, Näätänen R (1992). Intermodal selective attention. II. Effects of attentional load on processing of auditory and visual stimuli in central space. Electroencephalogr Clin Neurophysiol.

[27] Pakarinen S (2014). Fast determination of MMN and P3a responses to linguistically and emotionally relevant changes in pseudoword stimuli. Neurosci Lett..

[28] Kushnerenko E, Ceponiene R, Balan P, Fellman V, Näätänen R (2002). Maturation of the auditory change detection response in infants: a longitudinal ERP study. Neuroreport..

[29] Kuhl PK, Coffey-Corina S, Padden D, Dawson G (2005). Links between social and linguistic processing of speech in preschool children with autism: behavioral and electrophysiological measures. Dev Sci..

[30] Linnavalli T, Putkinen V, Huotilainen M, Tervaniemi M (2017). Phoneme processing skills are reflected in children’s MMN responses. Neuropsychologia..

[31] Pesonen A-K (2013). Cognitive ability and decline after early life stress exposure. Neurobiol Aging..

[32] Harvison KW, Molfese DL, Woodruff-Borden J, Weigel RA (2009). Neonatal auditory evoked responses are related to perinatal maternal anxiety. Brain Cogn..

[33] Hunter SK (2012). Antidepressants may mitigate the effects of prenatal maternal anxiety on infant auditory sensory gating. Am J Psychiatry.

[34] van den Heuvel MI (2015). Maternal mindfulness and anxiety during pregnancy affect infants’ neural responses to sounds. Soc Cogn Affect Neurosci.

[35] Karlsson L (2018). Cohort Profile: The FinnBrain Birth Cohort Study (FinnBrain). Int J Epidemiol.

[36] Partinen M, Gislason T (1995). Basic Nordic Sleep Questionnaire (BNSQ): a quantitated measure of subjective sleep complaints. J Sleep Res.

[37] Cox JL, Holden JM, Sagovsky R (1987). Detection of Postnatal Depression. Br J Psychiatry.

[38] Gibson J, McKenzie-McHarg K, Shakespeare J, Price J, Gray R (2009). A systematic review of studies validating the Edinburgh Postnatal Depression Scale in antepartum and postpartum women. Acta Psychiatr Scand.

[39] Rubertsson C, Börjesson K, Berglund A, Josefsson A, Sydsjö G (2011). The Swedish validation of Edinburgh Postnatal Depression Scale (EPDS) during pregnancy. Nord J Psychiatry.

[40] Kostilainen K (2018). Healthy full-term infants’ brain responses to emotionally and linguistically relevant sounds using a multi-feature mismatch negativity (MMN) paradigm. Neurosci Lett..

[41] Ross RG (2010). Research review: Cholinergic mechanisms, early brain development, and risk for schizophrenia. J Child Psychol Psychiatry.

[42] Zhao D (2017). Pharmacologic activation of cholinergic alpha7 nicotinic receptors mitigates depressive-like behavior in a mouse model of chronic stress. J Neuroinflammation.

[43] Adams CE, Yonchek JC, Zheng L, Collins AC, Stevens KE (2008). Altered hippocampal circuit function in C3H alpha7 null mutant heterozygous mice. Brain Res..

[44] Mennes M, Van den Bergh B, Lagae L, Stiers P (2009). Developmental brain alterations in 17 year old boys are related to antenatal maternal anxiety. Clin Neurophysiol..

[45] Buss C, Davis EP, Muftuler LT, Head K, Sandman CA (2010). High pregnancy anxiety during mid-gestation is associated with decreased gray matter density in 6-9-year-old children. Psychoneuroendocrinology..

[46] Clavarino AM (2010). Maternal Anxiety and Attention Problems in Children at 5 and 14 Years. J Atten Disord.

[47] Van den Bergh BRH, Marcoen A (2004). High antenatal maternal anxiety is related to ADHD symptoms, externalizing problems, and anxiety in 8- and 9-year-olds. Child Dev..

[48] Davis EP, Sandman CA (2010). The timing of prenatal exposure to maternal cortisol and psychosocial stress is associated with human infant cognitive development. Child Dev.

[49] Barclay NL, Gregory AM (2013). Quantitative genetic research on sleep: a review of normal sleep, sleep disturbances and associated emotional, behavioural, and health-related difficulties. Sleep Med Rev.

[50] Hedman C, Pohjasvaara T, Tolonen U, Suhonen-Malm A, Myllylä V (2002). Effects of pregnancy on mothers’ sleep. Sleep Med..

[51] Friederici AD, Friedrich M, Weber C (2002). Neural manifestation of cognitive and precognitive mismatch detection in early infancy. Neuroreport..

[52] Suppiej A (2010). Auditory processing during sleep in preterm infants: An event related potential study. Early Hum Dev.

[53] Cheour-Luhtanen M (1996). The ontogenetically earliest discriminative response of the human brain. Psychophysiology..

[54] Sambeth A (2009). Change detection in newborns using a multiple deviant paradigm: a study using magnetoencephalography. Clin Neurophysiol..

[55] Leppänen PHT (2004). Maturational effects on newborn ERPs measured in the mismatch negativity paradigm. Exp Neurol..

